# Formononetin Regulates Multiple Oncogenic Signaling Cascades and Enhances Sensitivity to Bortezomib in a Multiple Myeloma Mouse Model

**DOI:** 10.3390/biom9070262

**Published:** 2019-07-07

**Authors:** Chulwon Kim, Jong Hyun Lee, Jeong-Hyeon Ko, Arunachalam Chinnathambi, Sulaiman Ali Alharbi, Omar H.M. Shair, Gautam Sethi, Kwang Seok Ahn

**Affiliations:** 1 College of Korean Medicine, Kyung Hee University, 24 Kyungheedae-ro, Dongdaemun-gu, Seoul 02447, Korea; 2 Department of Botany and Microbiology, College of Science, King Saud University, Riyadh 11451, Saudi Arabia; 3 Department of Pharmacology, Yong Loo Lin School of Medicine, National University of Singapore, Singapore 117600, Singapore

**Keywords:** Formononetin, bortezomib, NF-κB, PI3K/AKT, AP-1

## Abstract

Here, we determined the anti-neoplastic actions of formononetin (FT) against multiple myeloma (MM) and elucidated its possible mode of action. It was observed that FT enhanced the apoptosis caused by bortezomib (Bor) and mitigated proliferation in MM cells, and these events are regulated by nuclear factor-κB (NF-κB), phosphatidylinositol 3-kinase (PI3K)/AKT, and activator protein-1 (AP-1) activation. We further noted that FT treatment reduced the levels of diverse tumorigenic proteins involved in myeloma progression and survival. Interestingly, we observed that FT also blocked persistent NF-κB, PI3K/AKT, and AP-1 activation in myeloma cells. FT suppressed the activation of these oncogenic cascades by affecting a number of signaling molecules involved in their cellular regulation. In addition, FT augmented tumor growth-inhibitory potential of Bor in MM preclinical mouse model. Thus, FT can be employed with proteasomal inhibitors for myeloma therapy by regulating the activation of diverse oncogenic transcription factors involved in myeloma growth.

## 1. Introduction

Multiple myeloma (MM) is a bone marrow based severe malignancy, which is extremely difficult to cure [1,2,3,4,5]. Multiple myeloma is the second most common blood cancer comprising around 10% of all hematological malignancies [6,7,8]. Although various pharmacological strategies have been developed to cure myeloma patients, including the application of proteasome inhibitors, immunomodulatory agents, and alkylating agents, there still remains an unmet need to improve the therapeutic outcome for myeloma patients [9,10,11].

Nuclear factor-κB (NF-κB) and activator protein-1 (AP-1) are major transcription factors implicated in the regulation of inflammation, immunomodulation, angiogenesis, and tumorigenesis [12,13,14,15]. It is made up of p50 and p65 subunits, and these are kept in a dormant state by a family of inhibitory proteins consisting of Inhibitory Subunit of NF Kappa B Alpha (IκBα), IκBβ, and other members [13,16]. Generally, NF-κB in the inactive form is made up of p50, p65, and IκBα and resides in the cytoplasm [17]. However, processing of IκBα can cause p50-p65 dimer to move into the nucleus, bind to the DNA, and modulate transcription [14,18,19]. AP-1 is also mainly composed of Jun, Fos, and Activating transcription factor (ATF) protein dimers [20]. Two transcription factors can regulate proinflammatory gene products in response to cytokines, growth factors, stress signals, bacterial, and viral infections, as well as oncogenic stimuli [21]. Interestingly, NF-κB pathway is often deregulated in myeloma cells and actively contributes to their survival and tendency to develop resistance against anti-myeloma drugs [22,23]. NF-κB and AP-1 are also important for osteoclastogenesis, which is relevant in myeloma, given the associated bone loss associated with this malignancy [24,25].

In addition to hyperphosphorylation of NF-κB pathway, myeloma cells have been documented to display activated phosphatidylinositol 3-kinase (PI3K) cascade that can regulate proliferative and anti-apoptotic properties of these tumor cells [22,26,27,28,29,30]. Besides these two aberrant signaling cascades, serine-threonine mitogen-activated protein kinases (MAPKs) encompassing extracellular signal-related kinases (ERKs), c-jun NH2-terminal kinases (JNKs), and p38 MAPKs can also regulate myeloma progression [31,32,33,34]. Additionally, AP-1 can induce MM cell proliferation, survival, and drug resistance within the bone marrow microenvironment [35]. Thus, the development of novel pharmacological strategies to simultaneously target multiple oncogenic cascades can unravel novel treatment options for myeloma patients.

The identification and development of small molecules from existing natural sources can be an effective approach for therapeutically targeting various malignancies, including myeloma [36,37]. Formononetin (7-hydroxy-3-(4-methoxyphenyl)chromen-4-one) (FT), an isoflavone, predominantly isolated from the roots of *Astragalus membranaceus*, *Trifolium pratense*, *Glycyrrhiza glabra*, and *Pueraria lobate* can affect various important hallmarks of cancer in different malignant cells by diverse molecular mechanism(s) [38,39,40,41,42,43]. It can display numerous pharmacological properties, including anti-inflammatory, antioxidant, antiviral and neuroprotective activity, and wound healing capability [38,44,45,46,47]. Moreover, FT was reported recently by our group to affect myeloma growth through the negative regulation of signal transducer and activator of transcription (STAT) 3/5 cascades mediated via oxidative stress [48]. However, the possible action of FT on NF-κB, PI3K/AKT, and AP-1 signaling pathways [35,49,50] has not been investigated before. Here, we analyzed whether FT can exert its anti-neoplastic actions via the modulation of the NF-κB, PI3K/AKT, and AP-1 signaling axis in myeloma model. The potential of FT to enhance the anti-tumoral activity of bortezomib (Bor), a targeted agent used for myeloma treatment, was further deciphered in a xenograft mouse model.

## 2. Materials and Methods

### 2.1. Reagents and Cell Lines

Formononetin was from Selleck Chemicals (Houston, TX, USA). 3-(4,5-dimethylthiazol-2-yl)-2,5-diphenyltetrazolium bromide (MTT), propidium iodide (PI), Tris base, glycine, NaCl, sodium dodecyl sulfate (SDS), and bovine serum albumin (BSA) were purchased from Sigma-Aldrich (St. Louis, MO, USA). p-IκB kinase (IKK)α/β, IKKα/β, p-IκBα, p-PI3K(Tyr458), PI3K, p-AKT(Ser473), p-p38(Thr180/Tyr182), p38, p-ERK1/2(Thr202/Tyr204), ERK1/2, p-JNK(Thr183/Tyr185), JNK antibodies were purchased from Cell Signaling Technology (Beverly, MA, USA). AKT, Bcl-2, Bcl-xL, Survivin, (Inhibitor of apoptosis proteins)IAP-1, IAP-2, (Cyclooxygenase)COX-2, Ki-67, (Matrix Metalloproteinase)MMP-9, Vascular Endothelial Growth Factor (VEGF), Caspase-3, Poly (ADP-ribose) polymerase (PARP), IκBα, p-p65, p65, c-Fos, c-Jun, p-p53, p53, p21, β-actin, Lamin B antibodies were from Santa Cruz Biotechnology (Santa Cruz, CA, USA).

Human multiple myeloma cell lines U266 and RPMI 8226 were obtained from the American Type Culture Collection (Manassas, VA, USA). U266 and RPMI 8226 cells were cultured in RPMI 1640 medium containing 10% FBS supplemented with 100 U/mL of penicillin and 100 μg/mL of streptomycin. 

### 2.2. Western Blotting

After the cells were treated with the indicated concentrations of FT, Western blot analysis was done as elaborated upon previously [33]. Briefly, cell lysates were separated by SDS-PAGE and transferred onto a nitrocellulose membrane. The membrane was blocked and probed with various antibodies. The proteins were detected with enhanced chemiluminescence (Millipore, Bedford, MA, USA). The bands were quantified using an Image J software (v1.8.0, National Institutes of Health, Bethesda, MD, USA). 

### 2.3. Electrophoretic Mobility Shift Assay for NF-κB and AP-1 -DNA Binding

The binding of NF-κB- and AP-1-DNA was analyzed by electrophoretic mobility shift assay (EMSA) as described before in both tumor cell lines and tissues [48]. Briefly, the nuclear extract was incubated with 5′-biotinylated NF-κB (5′-AGTTGAGGGGACTTTCCCAGGC-3′ and 5′-GCCTGGAAAGTCCCCTCAACT-3′), 5′-biotinylated AP-1 (5′-CGCTTGATGAGTCAGCCGGAA-3′ and 5′-TTCCGGCTGACTCATCAAGCG-3′), and Oct-1 (5′-TTCTAGTGATTTGCATTCGACA-3′ and 5′-TGTCGAATGCAAATCACTAGAA-3′) oligonucleotide probe. The protein-DNA complex was then separated on a 5% native polyacrylamide gel and transferred onto a nylon membrane and detected using the LightShift^®^ Chemiluminescent EMSA kit (Thermo Fisher Scientific Inc., Waltham, MA, USA). 

### 2.4. Reverse Transcription Polymerase Chain Reaction (RT-PCR)

Total RNA was extracted according to the manufacturer’s instructions (Invitrogen, Life Technologies, Carlsbad, CA, USA), and RT-PCR was carried out as indicated before [33]. 

### 2.5. Immunocytochemistry for p65, c-Fos, and c-Jun Localization

After the U266 cells were treated with 100 µM of FT, immunocytochemistry for various proteins was performed as described before [48]. 

### 2.6. TUNEL Assay 

U266 and RPMI 8226 cells were subjected to Terminal deoxynucleotidyl transferase dUTP nick end labeling (TUNEL) staining, as described earlier [48]. To determine the effect of FT on the late apoptotic cell death, cells were quantified using TUNEL assay kit (Roche Diagnostics GmbH, Penzberg, Germany) and then analyzed by flow cytometry using FACScan Calibur flow cytometer and CellQuest software (Version 4.0, BD Biosciences, Becton-Dickinson, Franklin Lakes, NJ, USA). 

### 2.7. MTT Assay

Cell viability was measured by an MTT assay to detect NADH-dependent dehydrogenase activity as done before [33]. Drug combinations were evaluated using CalcuSyn (Version 2.0, BIOSOFT, Ferguson, MO, USA) software based on the multiple drug-effect equation of Chou-Talalay.

### 2.8. Flow Cytometry

The effect of FT on cell cycle distribution was determined using flow cytometry following staining with Propidium iodide (PI). Cells were treated with 50 µM of FT and 10 nM of Bor for 24 h. The cells were then collected and washed with PBS, fixed in 70% cold ethanol at 4 °C overnight. After addition of 25 µg/mL of PI to the cells for 30 min in the dark, the apoptotic Sub-G1 cell population was analyzed on a FACScan Calibur flow cytometer and CellQuest software (BD Biosciences, Becton-Dickinson, Franklin Lakes, NJ, USA).

### 2.9. Animals

All procedures involving animals were reviewed and approved by KHU Institutional Animal Care and Use Committee [KHUASP(SE)-17-110]. All the in vivo experiments were conducted strictly in accordance with the institutional guidelines and monitored regularly. Five-week-old athymic nu/nu female mice (NARA Biotech, Korea) were implanted subcutaneously in the right flank with U266 cells. Tumors were allowed to grow to a maximum diameter of 1 to 1.5 cm and were then sacrificed.

### 2.10. Experimental Protocol

U266 cells [1 × 10^7^/100 μL PBS:Matrigel (1:1)] were injected subcutaneously into the right flank of the mice, as described previously [51]. When tumors have reached 0.5 cm in diameter, the mice were randomized into four treatment groups (*n* = 5/group). Group I was given PBS (200 μL, i.p. thrice/week), group II was given FT (20 mg/kg body weight, i.p. thrice/week), group III was given Bor (0.25 mg/kg body weight, i.p. thrice/week), group IV was given FT (20 mg/kg body weight, i.p. thrice/week) and Bor (0.25 mg/kg body weight, i.p. once/week). Therapy was continued for 21 days, and the animals were euthanized one week later. Primary tumors were excised, and the final tumor volume was measured as V = 4/3 πr^3^, where r is the mean radius of the three dimensions (length, width, and depth), and thereafter tissues were processed as described before [48].

### 2.11. Immunohistochemical and Western Blot Analysis of Multiple Myeloma Tumor Samples

Immunohistochemical and Western blot analysis for tumor samples was done as described previously [48].

### 2.12. Statistical Analysis

All numeric values are represented as the mean ± standard error (SE). Statistical significance of the data was determined by GraphPad Prism version 5 (GraphPad Software, La Jolla, CA, USA) using one-way ANOVA followed by Tukey’s posthoc test. Significance was set at *p* < 0.05.

## 3. Results

### 3.1. Formononetin Inhibits NF-κB and AP-1 Activation in Multiple Myeloma Cells

**Figure 1:**
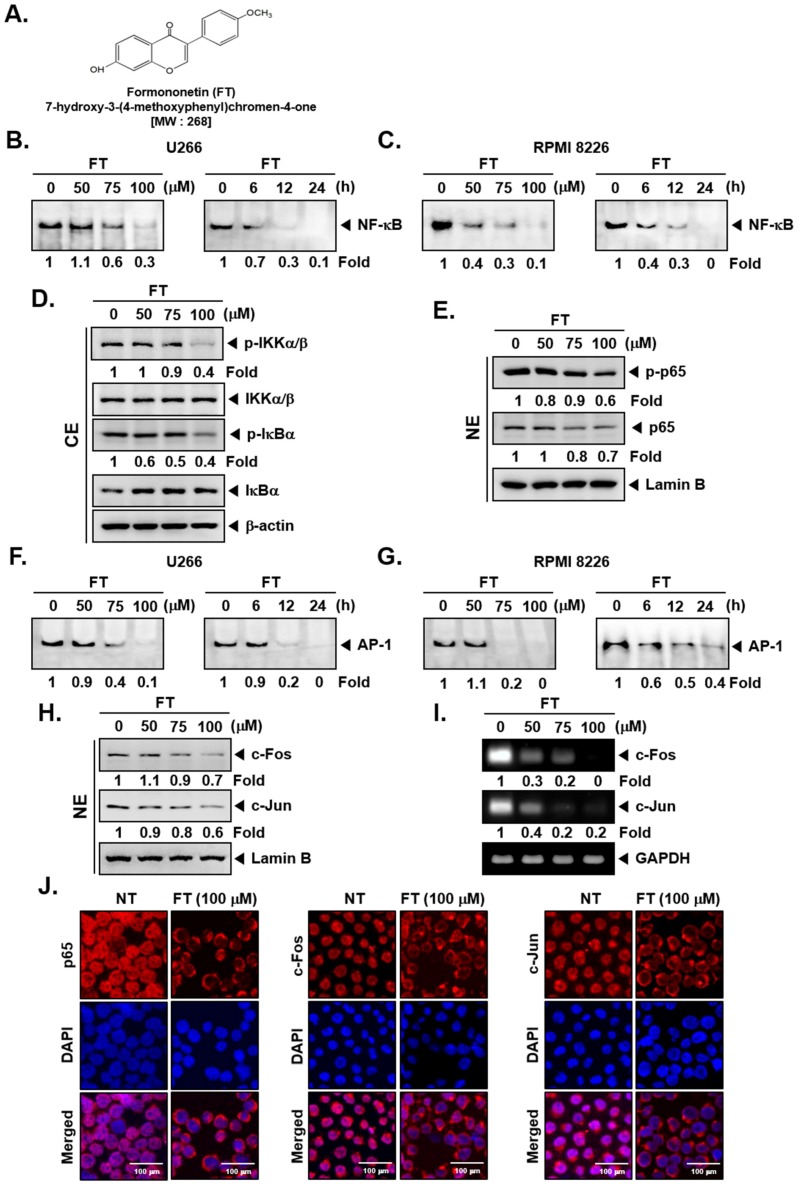
Formononetin (FT) affects nuclear factor-κB (NF-κB) and activator protein-1 (AP-1) activation in multiple myeloma (MM) cells. (**A**) The chemical structure of FT. (**B**,**C**) FT suppresses NF-κB binding activity in U266 and RPMI 8226 cells. Cells were exposed to the indicated concentrations of FT for 6 h (*left panel*) and treated with 100 μM of FT for various time intervals (*right panel*), analyzed for nuclear NF-κB levels by electrophoretic mobility shift assay (EMSA). (**D**,**E**) U266 cells (1 × 10^6^ cells/well) were incubated with indicated concentrations of FT for 6 h, and Western blot was carried out. (Uncropped gel images of **D** are provided in Supplementary Figure S1). (**F**,**G**) FT suppresses AP-1 binding activity. Cells were treated as described above in B, and EMSA was performed. U266 cells were treated as described above, and (**H**) Western blotting and (**I**) RT-PCR were done. (**J**) U266 cells were treated as described above, and immunostaining was carried out. The third panel shows the merged images of the first and second panels. The results shown are representative of three independent experiments. For band density, densitometric analysis was performed using an Image J software, and numbers on the bottom of the bands represent fold change in expression level relative to controls. NT: non-treated; CE: cytoplasmic extract; NE: nuclear extract.

NF-κB activation can correlate with the resistance to apoptotic cell death and also regulates the tumorigenic process in various hematological malignancies, including multiple myeloma [52,53,54]. Hence, we investigated whether FT has the potential to inhibit NF-κB activation, and consequently causing programmed cell death. The structure of FT is shown in Figure 1A. Cytoplasmic (CE) and nuclear extract (NE) were generated from myeloma cells exposed to FT and examined for various assays. First, in NE, we assessed NF-κB activity by performing EMSA assay and noted that FT substantially reduced NF-κB-DNA binding ability (Figure 1B,C). We deciphered the action of FT on the activation of IKK that is required for IκBα phosphorylation. CEs were prepared and assayed by Western blot. As shown in Figure 1D and Supplementary Figure S1, FT abrogated the activation of IKKα/β in U266 cells with no effect on the IKKα/β levels (first and second panel). Moreover, FT effectively reduced the IκBα phosphorylation with minimal effect on IκBα protein levels (Figure 1D, third and fourth panel). Additionally, the levels of p-p65 and p65 were inspected in NEs using Western blot. FT mitigated the levels of p-p65 and p65 in MM cells (Figure 1E).

To determine the effects of FT on the activation of another oncogenic transcription factor, AP-1 [55], EMSA was again performed. As shown in Figure 1F,G, FT substantially attenuated AP-1-DNA binding activities that also led to the reduction in the protein (Figure 1H) and mRNA (Figure 1I) levels of c-Jun and c-Fos. Moreover, as shown in Figure 1J, immunocytochemistry data clearly demonstrated that FT also reduced the translocation of p65, c-Fos, and c-Jun into the nuclear compartment.

### 3.2. Formononetin Mitigates the Activation of PI3K/AKT and MAPK Pathways

**Figure 2:**
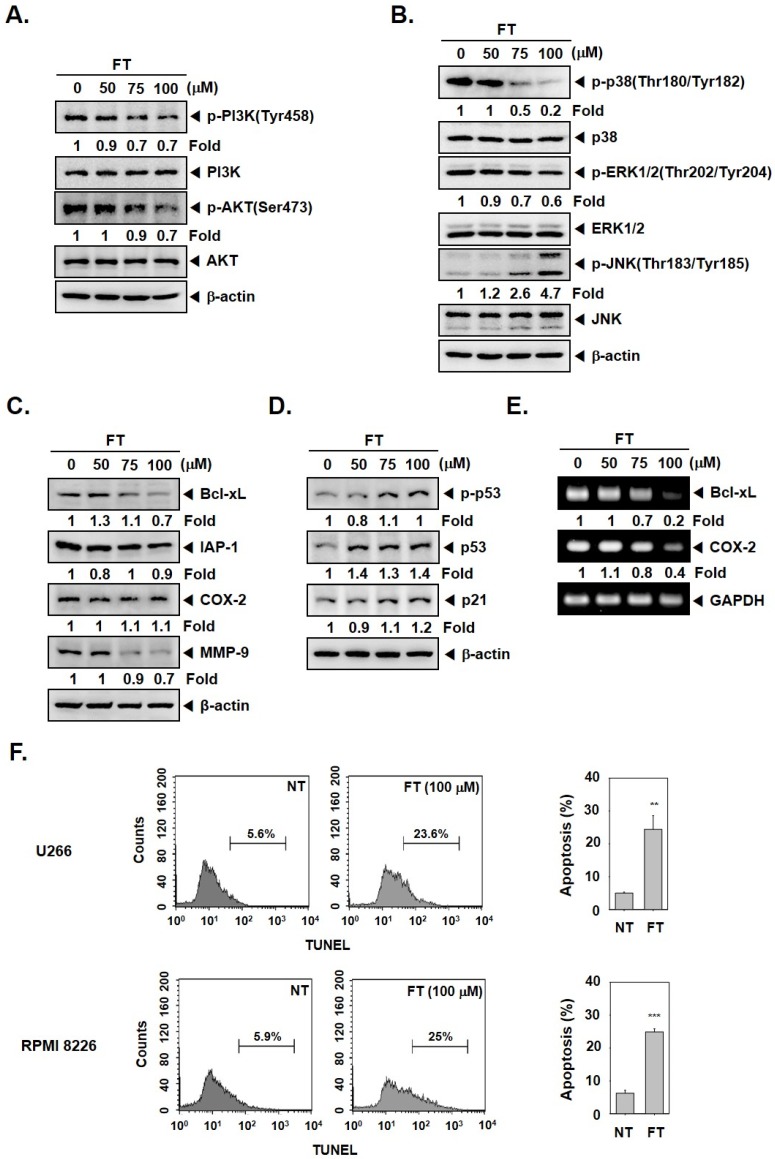
Formononetin (FT) regulates phosphatidylinositol 3-kinase (PI3K)/AKT/mitogen-activated protein kinase (MAPK) and induces apoptosis in multiple myeloma (MM) cells. (**A**,**B**) U266 cells (1 × 10^6^ cells/well) were incubated with indicated concentrations of FT for 6 h, and Western blot was done. (**C**,**D**) U266 cells (1 × 10^6^ cells/well) were treated with various indicated concentrations of FT for 24 h, and Western blot was performed. (**E**) U266 cells were treated with various indicated concentrations of FT for 24 h, and RT-PCR was performed. For band density, densitometric analysis was performed using an Image J software, and numbers on the bottom of the bands represent fold change in expression level relative to controls. (**F**) U266 and RPMI 8226 cells were treated with 100 μM of FT for 24 h, and TUNEL staining was done. The results shown here are representative of three independent experiments. ** *p* < 0.01, *** *p* < 0.001, vs. NT (non-treated).

We next deciphered if FT could also alter the phosphorylation of PI3K/AKT, which can also regulate aberrant tumor growth. As shown in Figure 2A, PI3K, as well as AKT phosphorylation, was affected by FT. Interestingly, it was discovered that FT also altered the levels of another important set of tumor-promoting proteins, namely, p-p38(Thr180/Tyr182), p-ERK1/2(Thr202/Tyr204), and p-JNK(Thr183/Tyr185), in tumor cells (Figure 2B).

### 3.3. Formononetin Downregulates the Levels of Tumorigenic Proteins and Causes Apoptosis

The survival proteins (Bcl-xL and IAP-1) can mediate resistance to apoptosis and drive the process of carcinogenesis [56]; hence, the effect of FT on these proteins was examined. The levels of Bcl-xL and IAP-1 in U266 cells were attenuated by FT exposure (Figure 2C,E). We deciphered the actions of FT also on the constitutive levels of COX-2 and MMP-9 proteins that can regulate the invasive ability of tumor cells. As depicted in Figure 2C,E, FT exposure induced a notable reduction in the levels of COX-2 and MMP-9. Figure 2D shows that FT can also augment the protein levels of p53 and p21, and the results of TUNEL assay further confirmed that FT could induce notable apoptosis in myeloma cells (Figure 2F).

### 3.4. Formononetin Causes Potentiation of the Anti-Tumorigenic Actions of Bortezomib

**Figure 3:**
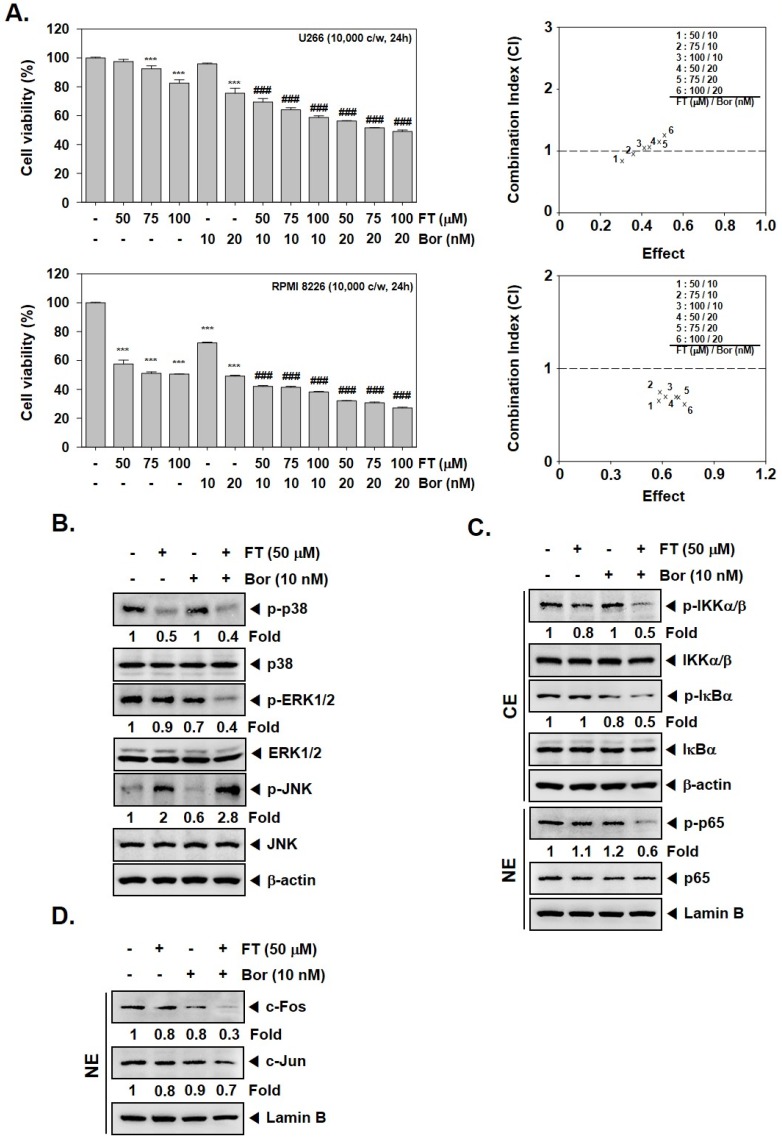
Formononetin (FT) enhances the anticancer effects of bortezomib (Bor). (**A**) U266 and RPMI 8226 cells (1 × 10^4^ cells/well) were treated with the indicated concentration of FT and Bor for 24 h. The cytotoxicity was determined by MTT assays, and the combination index (CI) values were obtained (*right panel*). (**B**–**D**) U266 cells (1 × 10^6^ cells/well) were seeded onto 6-well plates, incubated at 37 °C with indicated concentrations of FT and Bor for 6 h. Thereafter, Western blot was carried out against various proteins. The results shown here are representative of three independent experiments. For band density, densitometric analysis was performed using an Image J software, and numbers on the bottom of the bands represent fold change in expression level relative to controls. *** *p* < 0.001, vs. control, ### *p* < 0.001, vs. Bor alone. CE: cytoplasmic extract; NE: nuclear extract.

We noted that FT could augment the cytotoxic effects of Bor against MM cells (U266 and RPMI 8226), and the combination index (CI) values suggested that FT (50 μM)-Bor (10 nM), as well as FT (75 μM)-Bor (10 nM) concentrations synergistically attenuated cellular growth (Figure 3A). This action was mediated by its ability to enhance the effects of Bor in reducing p-p38 and p-ERK1/2 levels, and also causing an upregulation of p-JNK expression (Figure 3B). We also observed that combination treatment mitigated the p-IKKα/β, p-IκBα, p-p65 (Figure 3C), and c-Fos and c-Jun proteins (Figure 3D) levels which might also explain the action of FT in elevating the cytotoxic effects of Bor.

### 3.5. Formononetin Enhances the Apoptotic Effects of Bortezomib by Diverse Mechanisms

**Figure 4:**
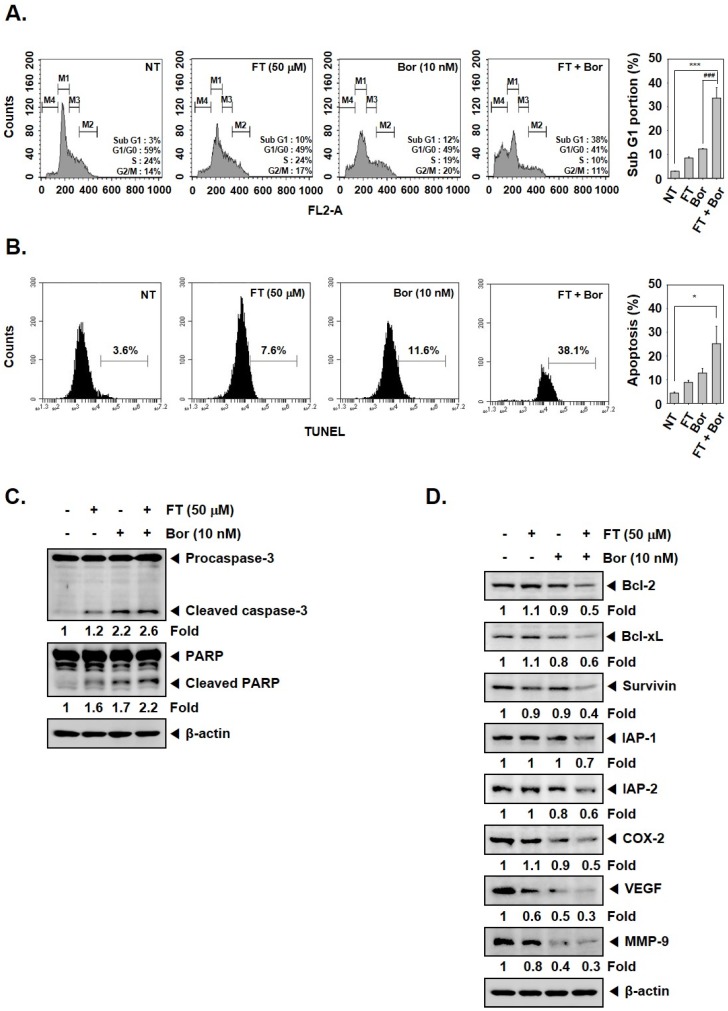
Formononetin (FT) and bortezomib (Bor) downregulates the expression of anti-tumorigenic proteins. (**A**) U266 cells (1 × 10^6^ cells/well) were treated with 10 nM of Bor in the presence and absence of 50 μM FT for 24 h, and the cells were washed, fixed, stained with Propidium Iodide (PI), and the apoptotic Sub-G1 cell population was analyzed by flow cytometry. M1, M2, M3, and M4 represent G1/G0, G2/M, S, and Sub-G1 phases, respectively. (**B**) U266 cells (1 × 10^6^ cells/well) were treated as indicated above, and the cells were stained with TUNEL and then analyzed for apoptotic effect by flow cytometry. (**C**,**D**) U266 cells (1 × 10^6^ cells/well) were exposed to the indicated concentrations of FT and Bor for 24 h, and Western blotting was done against various oncogenic proteins. The results shown here are representative of three independent experiments. For band density, densitometric analysis was performed using an Image J software, and numbers on the bottom of the bands represent fold change in expression level relative to controls. * *p* < 0.05, *** *p* < 0.001, vs. NT (non-treated), ### *p* < 0.001, vs. Bor alone.

As illustrated in Figure 4A, we noted that FT and Bor combination indeed caused the assembly of cells in sub-G1 stage up to 38%, and these findings were further confirmed by TUNEL assay (Figure 4B). Interestingly, the combination treatment not only escalated the levels of pro-apoptotic protein, caspase-3, as well as caused Poly (ADP-ribose) polymerase (PARP) cleavage (Figure 4C) but also mitigated the levels of various cancer-promoting proteins in myeloma cells (Figure 4D).

### 3.6. Formononetin Affects the Antitumor Actions of Bortezomib In Vivo

**Figure 5:**
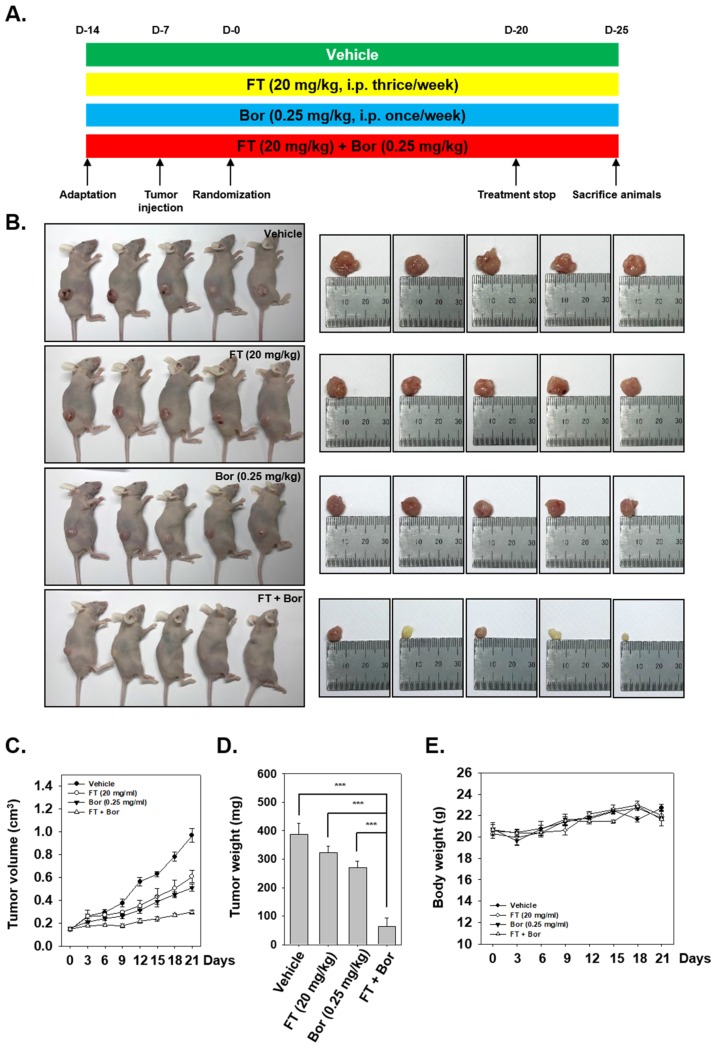
Effects of formononetin (FT) and bortezomib (Bor) on myeloma growth in vivo. (**A**) Schematic representation of experimental protocol. (**B**) Necropsy photographs of mice bearing subcutaneously implanted myeloma tumors. (**C**) Tumor volumes in mice measured during the course of the experiment. (**D**) Tumor weight was measured at the end of the experiment. (**E**) Body weight changes in FT and Bor treated mice. Data are a mean ± standard error (SE), *n* = 5. Both FT and Bor treatment resulted in a significantly lower tumor volume and weight compared to vehicle (*p* < 0.05, one-way ANOVA with Tukey post-test).

We examined the efficacy of both FT and Bor to affect tumor growth in the MM model based on protocol exhibited in Figure 5A. We noted that FT treatment alone, as well as in combination with Bor, significantly attenuated tumor growth and burden (Figure 5B–D) without affecting the body weight of the treated mice (Figure 5E).

### 3.7. Formononetin Affects the Levels of Oncogenic Biomarkers in Tumor Tissues

**Figure 6:**
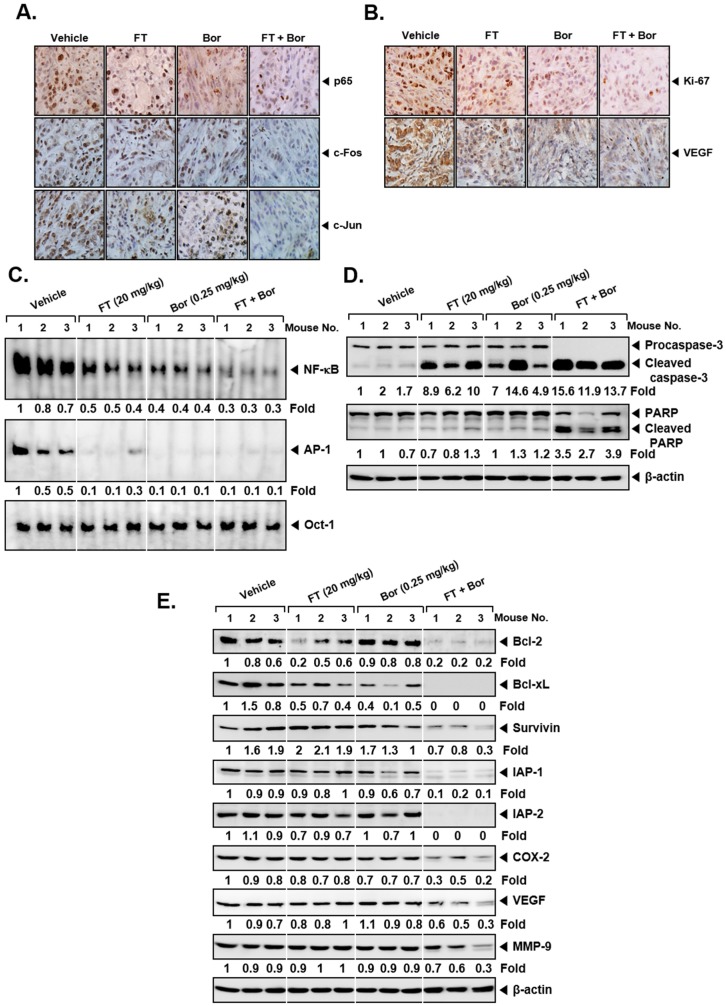
Combination of formononetin (FT) and bortezomib (Bor) effectively regulate diverse biomarkers in tumor tissues. (**A**) Immunohistochemical analysis of tumor tissues in U266 xenograft model after the treatment with antibodies of p65, c-Fos, and c-Jun (*left panels*). (**B**) Immunohistochemical analysis of Ki-67 (*right upper panels*) and Vascular endothelial growth factor (VEGF) (*right lower panels*). (**C**) EMSA of NF-κB and AP-1 in nuclear extracts from vehicle control, FT, and Bor treated mice tumor tissues. Oct-1 EMSA is shown as a loading control. (**D**,**E**) Western blot of caspase-3, PARP, B-cell lymphoma 2 (Bcl-2), Bcl-xL, Survivin, Inhibitor of apoptosis proteins-1 (IAP-1), IAP-2, COX-2, VEGF, and MMP-9 in lysates from vehicle control, FT, and Bor treated mice. Uncropped gel images for **E** are shown in Supplementary Figure S2. The results shown here are representative of three independent experiments. For band density, densitometric analysis was performed using an Image J software, and numbers on the bottom of the bands represent fold change in expression level relative to controls.

As observed in myeloma cell lines, we also noticed that combination treatment down-modulated the levels of p65, c-Fos, and c-Jun in tumor tissues analyzed (Figure 6A). In addition, a significant reduction in both Ki-67 and Vascular endothelial growth factor (VEGF) expression was noticed upon exposure to FT and Bor (Figure 6B), which indicated the efficacy of combination to mitigate the tumor growth and angiogenesis. Furthermore, combination treatment not only reduced DNA binding activities (Figure 6C) but also caused substantial apoptosis as observed in tumor tissues (Figure 6D, *first and second panels*). Also, it caused an attenuation in the levels of different biomarkers in tissues (Figure 6E and Supplementary Figure S2). Both FT and Bor were only marginally active when applied as single agents under in vivo settings.

## 4. Discussion

Targeting multiple signal transduction cascades (NF-κB, PI3K/AKT, and AP-1) can constitute an important pharmacological strategy as deregulation of these oncogenic pathways has been reported to mediate both initiation and progression of MM [33,53]. We noted that FT exerted substantial inhibitory effects on NF-κB, AP-1 (c-Fos and c-Jun), PI3K (tyrosine residue 458), and AKT (serine residue 473) activation. It was discovered that FT also augmented the apoptosis induced by a proteasomal blocker (Bor) and abrogated the growth of myeloma cells. FT also reduced the level of diverse tumorigenic proteins and enhanced the anti-tumor activity of Bor significantly in the preclinical model.

It was noticed that FT could suppress NF-κB and AP-1 in myeloma cells. It was noted that FT induced its suppressive action against NF-κB pathway by attenuating the phosphorylation of upstream IKK and thereby negatively regulating IκBα phosphorylation and nuclear translocation of p65 and its DNA binding affinity. Interestingly, Bor can regulate apoptosis of tumor cells by causing both inactivation as well as activation of NF-κB signaling machinery [57,58,59]. Moreover, in myeloma, an enhancement in the expression of receptor activator of nuclear factor kappa B ligand (RANKL) may lead to NF-κB and AP-1-mediated increased bone resorption [60,61]. In an interesting study, Bor was reported to abrogate both osteoclast differentiation as well as its bone resorption activity through the modulation of transcription factor-AP-1 [62]. Moreover, the activation of c-Fos and c-Jun can lead to tumorigenesis as well as resistance to chemotherapy. We noted that FT mitigated c-Fos and c-Jun activation at both protein and mRNA levels. Additionally, AP-1 DNA-binding affinity and nuclear translocation of c-Fos and c-Jun was also affected in myeloma cells.

The phosphorylation status of p38, ERK1/2, and JNK MAPKs was next examined in FT treated myeloma cells. It was noted that FT affected the activation of MAPKs by reducing the phosphorylation of p38 and ERK1/2, but enhancing the activation of JNK. Interestingly, the p38 pathway can be activated in monocytic precursors upon stimulating with different cytokines, and treatment with its pharmacological blocker SB203580 can lead to a reduced osteoclast differentiation [63,64]. On the contrary, JNK activation induced by a variety of mechanism(s), including the targeting of sphingolipid signaling, may regulate the survival of myeloma cells [65]. Furthermore, FT has been found to negatively affect the activation of PI3K/AKT cascade that can regulate ABC subfamily G member 2 (ABCG2) expression and mediate chemoresistance in myeloma cells [66], and we also observed a similar effect on PI3K/AKT axis in our experiments [66].

The negative regulation of AP-1 and NF-κB has been linked to the down-modulation of various oncogenic as well as metastatic proteins [67,68,69,70,71,72]. We noted that FT treatment caused a downregulation in the levels of diverse tumorigenic genes controlled by these transcription factors, thereby modulating the growth as well as inducing apoptosis in myeloma cells. It also caused an elevation in the levels of p53 and p21 gene products that can regulate both cell apoptosis and cell cycle progression. However, the direct mechanism of action of FT in tumor cells is still not clear from our present study, and the in silico docking analysis to decipher the possible interactions of FT with various proteins in different biochemical pathways will be carried out in future studies.

The application of Bor for myeloma therapy [73] can result in severe side effects and also drug resistance. Thus, overall, its effectiveness is greatly limited by its toxicity and development of chemoresistance [74]. The combination treatment of MM with Bor and other chemotherapeutic agents (e.g., doxorubicin and dexamethasone) has been used in clinical settings [75]. The strategy of employing natural products in combination with chemotherapy can form the basis of an important blueprint for treatment. We further demonstrated that the combination of FT and Bor treatment elicited a significantly greater antitumor effect in the preclinical model as compared with each treatment only. The combinatorial therapy used enhanced Bor-induced apoptosis through the downregulation of various pro-survival transcription factors and oncogenic proteins. In addition, our findings conclusively demonstrate that FT and Bor, when applied in conjunction, may exhibit significant anti-neoplastic effects in the preclinical model.

Overall, our findings in cell lines and in vivo model suggest that anti-tumoral actions of FT in myeloma model can be arbitrated via the regulation of multiple oncogenic cascades and gene products. Overall, the novel pharmacological combination of FT and Bor can be effectively used to supplement available treatment modalities for myeloma patients after completion of clinical trials.

## Supplementary Materials

The following are available online at https://www.mdpi.com/2218-273X/9/7/262/s1.
