# Supplementary material for: Formononetin Regulates Multiple Oncogenic Signaling Cascades and Enhances Sensitivity to Bortezomib in a Multiple Myeloma Mouse Model
Source: Biomolecules. 2019 Jul 7;9(7):262. doi: 10.3390/biom9070262 (PMC6681380; doi:10.3390/biom9070262)

Supplementary Figure 1: The whole gels for each of the cropped gel images shown in Figure 1D of the main text.

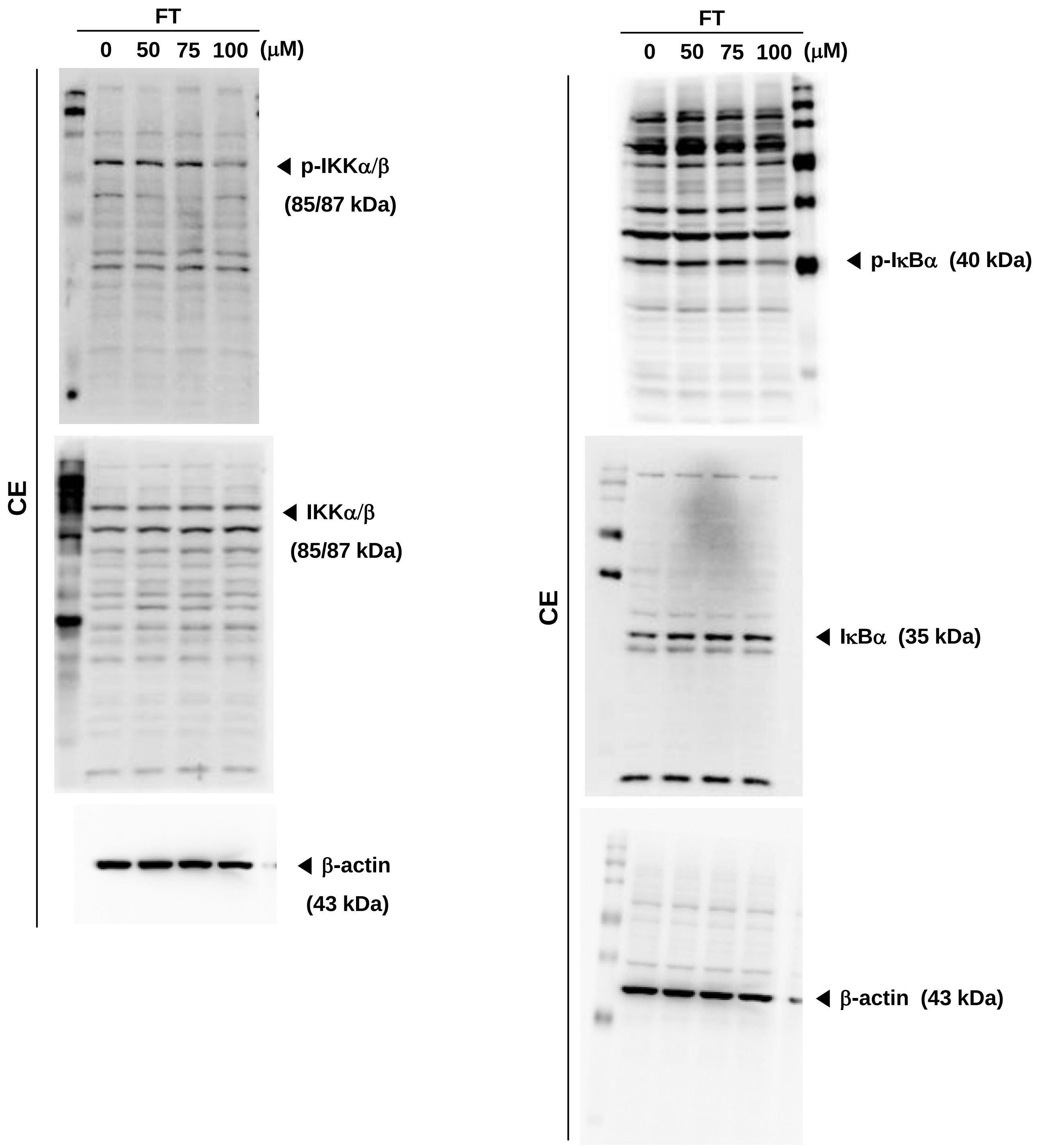

Supplementary Figure 2: The whole gels for each of the cropped gel images shown in Figure 6E of the main text.

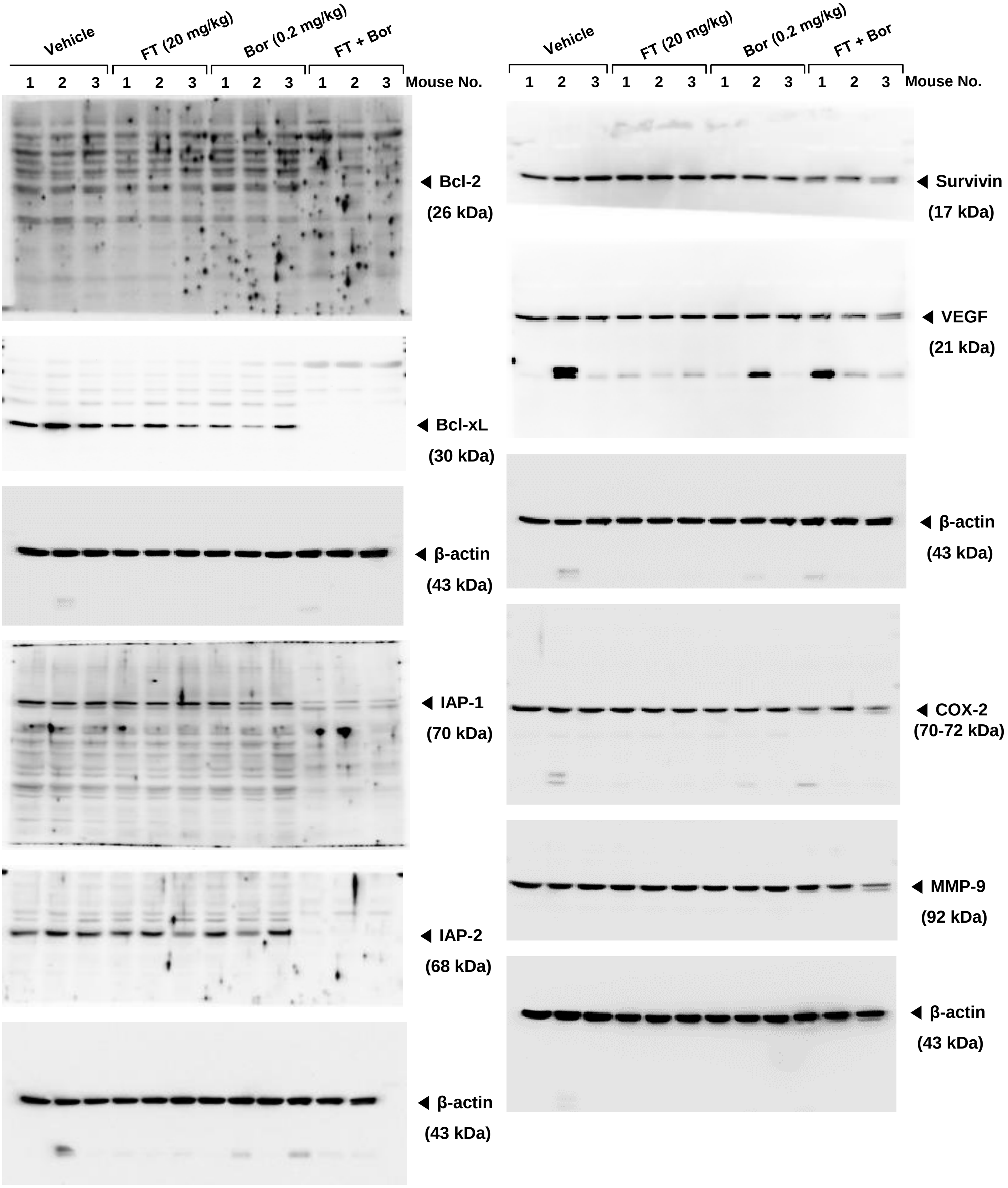

Supplement: Supplementary file 1 [file biomolecules-09-00262-s001.pdf]
